# Reconsolidation of traumatic memories protocol compared to trauma-focussed cognitive behaviour therapy for post-traumatic stress disorder in UK military veterans: a randomised controlled feasibility trial

**DOI:** 10.1186/s40814-023-01396-x

**Published:** 2023-10-13

**Authors:** J. Sturt, R. Rogers, C. Armour, D. Cameron, L. De Rijk, F. Fiorentino, T. Forbes, C. Glen, A. Grealish, J. Kreft, I. Meye de Souza, E. Spikol, V. Tzouvara, N. Greenberg

**Affiliations:** 1 Faculty of Nursing, Midwifery and Palliative Care, King’s College London, London, UK; 2 Research Centre for Stress, Trauma, and Related Conditions (STARC), School of Psychology, Queens University Belfast, Belfast, Northern Ireland; 3 Inspire Wellbeing, Lombard Street, Belfast, BT1 1RB Northern Ireland; 4 Institute of Psychiatry, Psychology and Neuroscience, King’s College London, London, UK; 5 Faculty Of Education & Health Sciences, University of Limerick, Limerick, Ireland; 6 Military Veterans’ Service, Pennine Care NHS Foundation Trust, Ashton-Under-Lyne, UK

**Keywords:** Post-traumatic stress disorder, PTSD, Veteran reconsolidation of traumatic memories, Trauma-Focussed CBT, Charity online therapy, PCL5

## Abstract

**Background:**

Post-traumatic stress disorder (PTSD) occurs more commonly in military veterans than the general population. Whilst current therapies are effective, up to half of veterans commencing treatment do not complete it. Reconsolidation of Traumatic Memories (RTM) protocol is a novel, easy to train, talking therapy with promising findings. We examine the feasibility of undertaking an efficacy trial of RTM in veterans.

**Methods:**

A parallel group, single-centre randomised controlled feasibility trial with a post-completion qualitative interview study. Sixty military veterans were randomised 2:1 to RTM (*n* = 35) or Trauma Focussed Cognitive Behaviour Therapy (CBT) (*n* = 25). We aimed to determine the rate of recruitment and retention, understand reasons for attrition, determine data quality and size of efficacy signal. We explored veterans’ perceptions of experiences of joining the trial, the research procedures and therapy, and design improvements for future veteran studies. Military veterans with a diagnosis of PTSD or complex PTSD, and clinically significant symptoms, were recruited between January 2020 and June 2021. Primary outcome was feasibility using pre-determined progression criteria alongside PTSD symptoms, with depression, recovery, and rehabilitation as secondary outcomes. Data were collected at baseline, 6, 12, and 20 weeks. Interviews (*n* = 15) were conducted after 20 weeks. Both therapies were delivered by trained charity sector provider therapists.

**Results:**

Participants’ mean age was 53 years, the mean baseline PTSD symptoms score assessed by the Post-traumatic Stress Checklist (PCL-5) was 57 (range 0–80). Fifty had complex PTSD and 39 had experienced ≥ 4 traumas. Data were analysed at 20 weeks for feasibility outcomes (*n* = 60) and mental health outcomes (*n* = 45). Seven of eight progression criteria were met. The RTM group experienced a mean 18-point reduction on the PCL-5. TFCBT group participants experienced a mean reduction of eight points. Forty-eight percent of the RTM group no longer met diagnostic criteria for PTSD compared to 16% in the TFCBT group. All veterans reported largely positive experiences of the therapy and research procedures and ways to improve them.

**Conclusion:**

RTM therapy remains a promising psychological intervention for the treatment of PTSD, including complex PTSD, in military veterans. With specific strengthening, the research protocol is fit for purpose in delivering an efficacy trial.

**Trial registration:**

ISRCTN registration no 10314773 on 01.10.2019.

Full trial protocol: available on request or downloadable at ISRCTN reg. no. 10314773.

## Key messages regarding feasibility

1. What uncertainties existed regarding the feasibility?
  Can United Kingdom (UK) military veterans be recruited, randomised and retained in a therapy trial?
  Will RTM have an efficacy signal in a UK context?
  Are there any safety events associated with RTM?
  Can charity therapists be trained to deliver RTM competently in a comparable timeframe as US evidence indicates?
2. What are the key feasibility findings?
  UK military veterans can be recruited, randomised and largely retained. Fewer women and ethnic minority participants were recruited.
  The RTM effect signal is strong compared to the comparison therapy.
  No safety events were observed.
  Charity therapists achieved RTM competency in the expected timeline.
3. What are the implications of the feasibility findings for the design of the main study?
  There is a need to strengthen and standardise eligibility criteria around therapy readiness for an efficacy trial. This can be undertaken at the diagnostic assessment interview.
  Recruitment of a more diverse sample is required. We will utilise public engagement and community outreach to adapt our recruitment strategy and target our clinical trial sites in more ethnically diverse populations. We will broaden our social media campaign by using more real-world representation.
  For a future pragmatic trial, the RTM therapy delivery platform within the NHS requires development.

## Background

Post-traumatic stress disorder (PTSD) is a mental health condition experienced by a minority of people who are exposed to traumatic events [2]. Whilst PTSD may resolve spontaneously, this is uncommon [2–6]. PTSD adversely affects social and occupational functioning and is associated with poor physical health and disruption of family and interpersonal relationships. Other evidence demonstrates PTSD is also linked with homelessness, substance misuse and suicidality [3–6]. Studies show that rates of PTSD are elevated in military veterans, with up to 17% of UK combat-exposed troops affected by the condition [7]. A recent study showed that around 37% of Northern Irish veterans (*n* = 1267) met criteria for PTSD [8] which is substantially higher than a general UK population prevalence rate of PTSD of 5% in any previous 12 months [9].

Furthermore, the relatively new diagnostic entity of Complex PTSD (CPTSD), characterised by a high symptom burden, appears particularly common amongst military veterans with studies showing that up to 80% of help-seeking veterans with PTSD also have CPTSD [9, 10]. This suggests that veterans with PTSD are a population vulnerable to severe mental ill health and poor quality of life.

Current evidence-based UK guidelines (NICE, 2018) recommend individuals who access care for PTSD receive either Trauma-Focussed Cognitive Behavioural Therapy (TFCBT) or Eye Movement Desensitisation and Reprocessing [EMDR] [3] typically between eight and twelve sessions for optimal efficacy [11]. However, in contrast to TFCBT, EMDR is not recommended for combat-related PTSD [3]. Training required for both therapies is extensive and time consuming. Importantly, non-response rates to TFCBT in veterans can be as high as 50% [3, 12]. Given the scale of the problem of PTSD in veterans, and due to the practical challenges of providing timely, evidence-based therapy for PTSD, there is a pressing need for accessible, cost-effective treatments for PTSD which could ideally be delivered in fewer sessions.

This paper examines the feasibility of using a novel treatment called Reconsolidation of Traumatic Memories (RTM) which has been assessed in several small-scale studies in the United States in veteran populations. Results show high completion rates (ranging from 87 to 100%) and low participant dropout [13–15]. Furthermore, after a mean of three sessions a rapid decline in self-reported PTSD symptoms and PTSD caseness in the Diagnostic Statistical Manual for Mental Disorders (DSM-5) was found [2]. Whilst these results are promising, RCT studies are needed to properly assess the value of RTM as a potentially cost-effective and acceptable treatment for PTSD in a UK veteran population. Furthermore, veterans are challenging to recruit, randomise, and retain into mental health treatment studies [16]. The PTSD Experimental Treatment Trial (PETT Study) aimed to determine the feasibility of a research protocol for evaluating RTM compared to TFCBT in military veterans in order to inform an efficacy trial.

## Methods

Our research objectives were fourfold: (1) determine the rate of trial recruitment, retention in treatment and research, understand reasons for drop out and determine completeness of outcome data assessed against progression criteria to determine if an efficacy trial is deliverable; (2) undertake exploratory analyses of the outcome data to support a power calculation for an efficacy trial; (3) understand the safety risks associated with RTM; and (4) explore veterans’ experiences of joining the trial, the research procedures and therapy, and how to improve the research design for future studies with a veteran population.

## Research design

We conducted a parallel group, single-centre feasibility randomised controlled trial with a post-trial qualitative interview study. Randomisation was stratified by (a) diagnosis of simple or complex PTSD (CPTSD), and (b) sex. The trial is registered on 01.10.2019 with ISRCTN reference 10314773. Ethical approval was granted by King’s College London Research Ethics approval reference HR-18/19–11320 on 19.04.2019.

### COVID-related changes to the protocol

Following registration of the original protocol, three COVID-19-related changes were made in consultation with the Trial Steering Group, the Data Monitoring and Ethics Committee and the KCL Ethics Committee. These were as follows: Initially randomisation was 1:1 ratio but changed at the recruitment mid-point to a 2:1 ratio favouring the experimental treatment arm. This resulted from COVID-19-related recruitment delays associated with lockdowns and reduced therapists’ capacity in the comparison treatment arm. Recruitment was widened from focusing solely on Northern Ireland to UK-wide veterans and the recruitment period was extended for an additional 6 months. In addition, delivery of both therapies necessarily moved online and the trial was paused for 6 weeks to enable the therapy provider to incorporate online therapy delivery on a secure platform and subsequently all therapies were delivered remotely via videocall. The methods and results presented reflect these changes.

### Participants and setting

Inclusion criteria were (1) adults ≥ 18 years, (2) UK military veterans from the Royal Navy, Army, Royal Air Force, (3) a diagnosis of PTSD determined by DSM-5 [2], (4) symptoms causing clinically significant distress or impact on social, occupational or other areas of functioning using the Clinician Administered PTSD scale (CAPS-5) [17] and the International Trauma Questionnaire (ITQ) [18], (5) living or working in the UK.

Exclusion criteria were (1) serving personnel, (2) currently receiving psychological treatment for PTSD, (3) a comorbid DSM-5 mental health or personality disorder sufficiently severe as to intrude upon the participant’s ability to cooperate with treatment, (4) dependence on alcohol, prescription medication or illegal substances, (5) suicidality within the previous month, (6) unable to provide informed consent, (7) self-reported Psychoactive medication changes in the previous 4 weeks, (8) other reason arising from eligibility assessment by Clinical Psychologists such as therapy readiness. The clinical elements of the trial were delivered via Inspire Wellbeing, an all-Ireland third sector organisation holding statutory public health contracts to treat and support people with mental health conditions, intellectual disabilities and addictions. They have extensive experience of working with veterans with complex needs as well as serving military and emergency service personnel routinely, unavoidably exposed to traumatic events.

### Participant recruitment and eligibility screening

Recruitment took place across the UK between February 2020 and June 2021. Veterans were informed of the trial through our charity clinical partner, Inspire Wellbeing, and their veteran mental health commissioning organisations, through our public engagement work with UK-wide veteran charities using traditional and online media announcements and through a targeted social media campaign. Potential participants contacted a dedicated PETT study email address or were referred from statutory and third sector veteran support agencies. After signing a GDPR compliant personal data processing consent form they completed the PTSD Checklist for DSM-5 (PCL-5) [19] to screen for eligibility. Veterans with a score ≥ 33 on the PCL-5, indicating probable PTSD, were invited to undergo the informed consent process and collection of baseline data. A PTSD and complex PTSD diagnostic interview was undertaken by a consultant clinical psychologist at Inspire using the Clinician Administered PTSD Scale (CAPS-5) [17] and the ICD-ITQ [18].

### Participant safety

Aligned with our research question regarding RTM safety, adverse and serious adverse events were operationally defined, monitored and risk escalation procedures put in place. RTM participants were provided with and regularly reminded of emergency telephone numbers along with the contact details for a trial-funded, but independent, trauma-experienced Clinical Psychologist. An adverse event was defined as a ≥ 10-point increase in the self-report PCL-5 in the interim between the previous therapy session, a 15-point rise from baseline or the maximum score of 80 being reached. Depression severity follow-up data assessed by the PHQ9 [20] were reviewed within 48 h of receipt to identify anyone at risk of self-harm or suicidality.

### Randomisation, stratification, and allocation concealment

King’s College London Clinical Trials Unit provided a computer randomisation system. Participants were randomised within 30 days of baseline assessment. Randomisation was stratified based on (1) diagnosis of PTSD or CPTSD and (2) sex. Eleven percent of the UK veteran population are of female sex and their military traumas can be different in origin [21]. Of these, 11% have PTSD with their traumas being different in origin from those of male veterans [22, 23]. We aimed to determine whether our research protocol would also attract female participants to progress equality, diversity, and inclusion. Unique participant IDs were generated and computer randomisation to therapy A or B occurred, and allocation sent to the only unblinded member of the research team and an administrator at Inspire for communication with the participant and allocation of therapist. The unblinded researcher supported the therapists’ data entry, monitored participant safety and had no contact with participants or their research data.

### Interventions and delivery

Ten Inspire therapists were invited to undertake professional training to deliver the interventions following completion of the revised Cognitive Therapy Scale [24] and providing current professional accreditation evidence and duration of experience of treating PTSD/CPTSD. We aimed to develop two therapy teams with comparable experience across 5 escalating levels. Level 1 therapists were newly accredited counsellors with limited experience of working with PTSD/CPTSD and competence self-reported across the majority of skills on the revised Cognitive Therapy Scale [24]. In contrast, level 5 had over 10 years post-accreditation experience, rated expert on the revised Cognitive Therapy Scale and had multiple professional accreditations. The two comparable teams were formed by randomly allocating each therapist according to level to join either of RTM or TFCBT training teams. RTM therapists undertook pre-course reading, 40 h over 4 days of face-to-face classroom teaching and 4 h of symptom assessment and therapy delivery on two trauma patients which were observed and assessed for fidelity by the RTM trainer/supervisor and an external assessor from the US research team. TFCBT therapists undertook 24 h of face-to-face classroom teaching aligned to Ehlers and Clark’s cognitive processing model [25, 26] incorporating reflective exercises alongside practical clinical case examples illustrating key intervention strategies. Their competency was assessed as the training progressed. Four therapists in the RTM arm and two in the TFCBT arm demonstrated competence and willingness to deliver the interventions within the trial. All therapies were delivered remotely on a secure online video platform by a single therapist who received therapy-specific clinical supervision within their therapy team.

#### Experimental RTM

The experimental RTM intervention [27], was delivered in two to four × 90-min sessions with at least one mandatory sleep cycle between sessions. The RTM Protocol is a brief cognitive intervention with minimal and non-traumatising exposure to the original stimulus. The manualised 89-step RTM protocol aims to rewrite the emotional elements of the memory by taking advantage of so-called reconsolidation [27]. Reconsolidation describes the reactivation of long term, otherwise permanent memories, by their evocation in certain contexts [28, 29]. When a memory is reactivated, it labilises, that is, it becomes subject to change. If the circumstances surrounding the memory remain the same, the memory remains unchanged; it is maintained in its current state. If circumstances have intensified, the impact of the memory may become worse; re-traumatization can add to the intensity of trauma memories. If the new circumstance provides evidence that a threat of negative emotional stimulus is no longer relevant, the strength of the affective charge may decrease. RTM was delivered over a 3- to 4-week period from first to final session.

#### Comparison TFCBT

TFCBT was delivered over up to 18 × 60 to 90-min weekly sessions [26]. The cognitive processing model of PTSD developed by Ehlers and Clark (2000) was purposely used in this study because it displays the largest treatment effect sizes and significant symptom improvement and is widely delivered through IAPT services [25, 26, 30]. TFCBT is focused on identifying the relevant appraisals, memory characteristics and triggers, and behavioural and cognitive strategies that maintain PTSD symptoms. TFCBT was delivered over an 18-week period from first to final session.

### Data collection

Data collection took place at baseline (Time (T) 1), and weeks 6 (T2), 12 (T3), and 20 (T4) post-randomisation. Questionnaires were completed by post, telephone or online using Qualtrics. Follow-up data was included if collected 10 days either side of the expected time point. Data was entered onto eCRF database (Elsevier MACRO) hosted on King’s Clinical Trial Unit encrypted server. Participants were offered a £15.00 shopping voucher when returning T2–4 questionnaires.

### Primary outcomes

These were feasibility related:

- Proportion recruited, defined as the number who consented to enter the study over the number who were screened for the study.
- Proportion randomised, defined as the number who were randomised to a treatment arm over the number who consented to enter the study.
- Proportion of drop out/research attrition, defined as the number who left the study over those who were randomised to a treatment arm.
- Completeness of outcome data, defined as the proportion of data which was complete at the 20-week outcome.

### Secondary outcomes

Mental health outcome data assessed data quality and was used to detect an effect signal and standard deviation to determine a sample size calculation for an efficacy trial. The anticipated primary outcome for a full trial is PTSD symptoms assessed by the PCL-5 [19]. A score of ≥ 33 is indicative of PTSD diagnosis. The minimal clinically important difference (MCID) for PCL-5 is a reduction in score of 10 points or more from baseline to 20 weeks [19]. The Work and Social Adjustment Scale (WSAS) [31] is a 5-item scale to assess the impact of the person’s mental health on work, home, social and private leisure activities and interpersonal relationships. A higher score is indicative of recovery. The MCID for WSAS is taken as a reduction in score of 8 points or more from baseline to 20 weeks [32]. The Quality of Process of Recovery scale (QPR) [33] assesses mental health recovery by measuring intrapersonal functioning and interpersonal functioning on a 0–88 scale with higher scores indicative of recovery. The Patient Health Questionnaire (PHQ) [20] is used as a screening instrument for depression. The PHQ-9 has a MCID of a reduction in score from baseline to 20 weeks of 5 points or more [32]. The General Anxiety Disorder (GAD 7) [34] screens for anxiety. Scores of 5, 10, and 15 are taken as the cut-off points for mild, moderate, and severe anxiety, respectively. The GAD MCID is a reduction in score from baseline to 20 weeks of 6 points or more [32]. The EQ VAS [35] assesses perceived health status on a 0–100 visual analogue scale.

### Sample size

We took into account recommendations for pilot trials which propose a method for determining an external pilot RCT sample size in order to estimate the sample size for the main RCT [36]. Trials comparing therapy and research attrition rates for TFCBT and EMDR in general PTSD populations found a range 8–58% with a mean of 29% [37]. Informed by these data we proposed screening 180 potential participants for eligibility and randomised 60 participants. See Table 1 for progression criteria relating to sample size.

**Table 1:** Pre-specified progression criteria to an efficacy trial

Project outcomes	Measure of success	No of participants
Outcome 1: Known rate of trial recruitment, retention in treatment, and research	In 14 months, we identify 180 eligible participants	180 study participants
	Consenting and randomised participants *n* = 60	60 study participants
	RTM treatment drop out ≤ 30% TFCBT treatment drop out ≤ 50%	≥ 36 study participants
	Research retention: 36 participants at 20 weeks	36 study participants
Outcome 2: Quality of outcome data	Baseline data complete for 90% of participants	54 study participants
	12-week data complete for 70% of participants	42 study participants
	20-week data complete for 50% of participants	30 study participants
Outcome 3: Known safety risks and ameliorations of RTM therapy	Adverse and serious adverse events and ameliorations recorded and discussed at the bi-weekly research team meeting	All 60 trial participants
	A log of every adverse, serious adverse event and clinical and research team actions in response	All 60 trial participants
Outcome 4: Establishment of expanded mental health care capacity in the veteran third sector	A minimum of 5 Inspire therapists will complete the 20-h training and be assessed as competent in delivering protocoled TFCBT	Ten Inspire therapists demonstrating competence in new therapeutic protocols and retained
	A minimum of 5 different Inspire therapists will complete RTM training and be assessed as competent in delivering the RTM protocol	
	Therapists attend two–four weekly clinical supervision sessions	All therapists

### Statistical methods

For the primary feasibility outcomes, raw numbers and proportions will be presented. The proportions are presented with the 95% CI. The analysis of the secondary outcomes aimed to estimate the mean and standard deviation for the PCL-5 score at 20 weeks for each treatment group and the mean difference and standard deviation of the in PCL-5 score from baseline up to 20 weeks for each treatment group. This method was repeated for the WSAS, QPR, PHQ-9, GAD-7, and EQ VAS data. Across the secondary outcomes, we present 95% CIs for the differences between arms.

### Trial management and oversight

A project management group of all investigators and the research team met on six occasions. The research teams from both King’s College London and Queen’s University Belfast met two-weekly to monitor recruitment, retention, and safety. The Trial Steering Committee comprised a consultant psychologist, a consultant forensic psychiatrist, and a charity representative, all of whom had veteran health expertise. The Data Monitoring and Ethics Committee comprised a consultant clinical psychologist, an independent statistician, a psychological therapist and a charity representative. These committees met jointly on three occasions. Participant safety was discussed at each meeting.

### Progression criteria to an efficacy trial

Criteria were agreed with the funder at the application stage according to important scientific trial criteria and strategic funding objectives of the funder (Table 1) using traffic light assessment.

### Qualitative study

Online semi-structured interviews aimed to (a) explore veterans’ experiences of joining the RCT, (b) their experiences of research procedures and therapy, and (c) their views on how to improve the research design for future studies with this population. Online semi-structured interviews were used as an adjunct to supplement and add depth to the trial results [38]. An interview guide was developed by the research team informed by current literature on RTM and the objectives of this study and structured into four main parts aligned with the objectives.

#### Qualitative data collection

Participants who had previously consented to an interview were contacted and participation in this phase advertised in our project newsletter. The sampling framework consisted of three groups: (a) veterans who completed RTM therapy, (b) veterans who completed TF-CBT therapy, and (c) those who did completed neither therapy. The interviews were conducted following the 20-week follow-up and immediately before the study end. Interviews lasted 40–60 min and were conducted via Zoom. The audio-recordings were saved to a King’s password protected laptop and were transcribed using Microsoft Word transcription function.

#### Qualitative data analysis

The six-step thematic analysis approach by Braun and Clarke was implemented to analyse and identify patterns of meaning [39]. Initial codes were generated and validated in coding teams and applied to remaining transcripts. A thematic map was generated to visually collate the codes under meaningful themes with names and definitions generated.

## Results

**Fig. 1:**
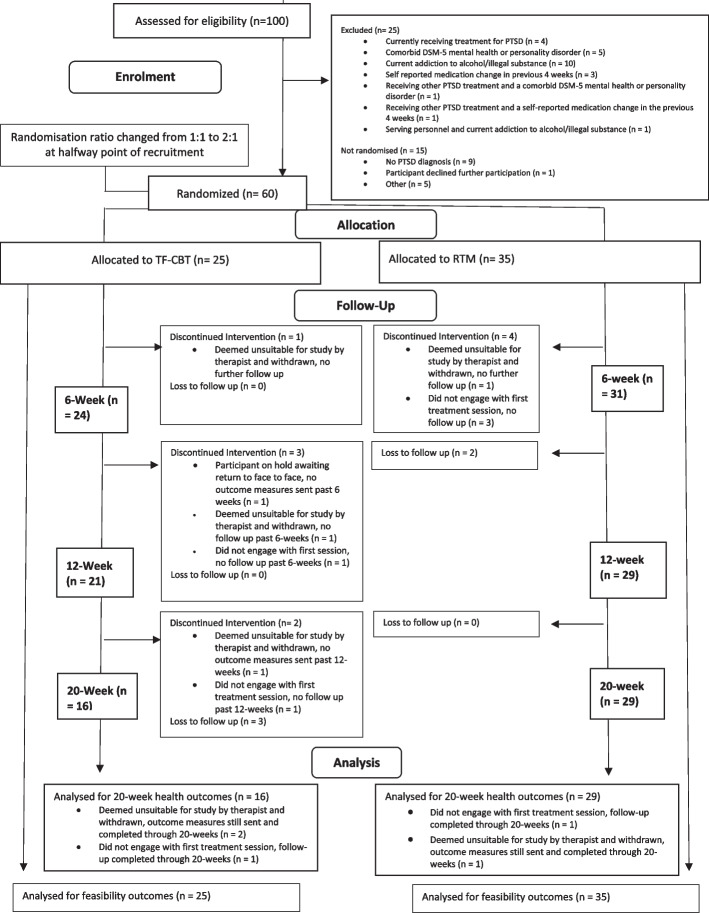
CONSORT flow diagram at 20 weeks (primary analysis timepoint)

One hundred participants were recruited between January 2020 and June 2021 with 60 randomised (see CONSORT diagram Fig. 1). Of the 100 participants assessed for eligibility, 75 had entered the study through engagement with the social media campaign.

Randomised participants (*N* = 60) had a mean age of 54 years (SD = 12); 55 (91%) were male, 56 (93%) were white British, 24 (40%) were not working and 40 (66%) were in a long-term relationship. All ranks and services were represented with greater proportions from the lower ranks, 46 (77%) had served for 5 years or more and 30 (50%) had been deployed overseas ≥ 3 times. The mean baseline PCL-5 score was 57 (SD = 11), 50 (83%) had complex PTSD and 39 (65%) had experienced ≥ 4 traumas. Full participant characteristics are detailed in Table 2.

**Table 2:** Randomised participant characteristics (overall and by arm)

Characteristics	TFCBT (*n* = 25)	RTM (*n* = 35)	Total (*n* = 60)
Age (years), *mean (SD)*	53.56 (11.79)	52.66 (8.01)	53.03 (9.68)
Age (years), *median (IQR)*	54 (46, 62)	52 (47, 57)	52 (46.5, 58.5)
Sex,* n (%)*			
Male	22 (88.0)	33 (94.3)	55 (91.7)
Female	3 (12.0)	2 (5.7)	5 (8.3)
Ethnicity, *n (%)*			
White	22 (88.0)	34 (97.1)	56 (93.3)
Any other ethnic group	1 (4.0)	1 (2.9)	2 (3.3)
Missing	2 (8.0)	0 (0.0)	2 (3.3)
Occupational status last 30 days, *n (%)*			
No paid work	10 (40.0)	14 (40.0)	24 (40.0)
Part-time paid work	2 (8.0)	6 (17.1)	8 (13.3)
Full time paid work	11 (44.0)	15 (42.9)	26 (43.3)
Missing	2 (8.0)	0 (0.0)	2 (3.3)
Relationship status,* n (%)*			
Long term relationship/married	13 (52.0)	27 (77.1)	40 (66.7)
Short term relationship	3 (12.0)	0 (0.0)	3 (5.0)
Not in a relationship	7 (28.0)	8 (22.9)	15 (25.0)
Missing	2 (8.0)	0 (0.0)	2 (3.3)
Living alone, *n(%)*			
No	14 (56.0)	24 (68.6)	38 (68.6)
Yes	8 (32.0)	10 (28.6)	18 (30.0)
Missing	3 (12.0)	1 (2.9)	4 (6.7)
Armed forces composition,* n (%)*			
Navy	0 (0.0)	3 (8.6)	3 (5.0)
Army	22 (88.0)	28 (80.0)	50 (83.3)
Royal Air Force	1 (4.0)	3 (8.6)	4 (6.7)
Missing	2 (8.0)	1 (2.9)	3 (5.0)
Rank on military exit,* n (%)*			
Lower rank^a^ (Pte to Cpl)	15 (60.0)	16 (45.7)	31 (51.7)
Senior rank^b^ (Sgt to WO1)	6 (24.0)	16 (45.7)	22 (36.7)
Officer rank	2 (8.0)	2 (5.7)	4 (6.7)
Missing	2 (8.0)	1 (2.9)	3 (5.0)
Duration of military service,* n (%)*			
≤ 4 years	4 (16.0)	8 (22.9)	13 (20.0)
5–12years	10 (40.0)	7 (20.0)	17 (28.3)
≥ 13 years	9 (36.0)	20 (57.1)	29 (48.3)
Missing	2 (8.0)	0 (0.0)	2 (3.3)
Times deployed overseas for 30 days or more,* n (%)*			
≤ 2 times	12 (48.0)	16 (45.7)	28 (46.7)
3–5 times	7 (28.0)	9 (25.7)	16 (26.7)
More than 5 times	4 (16.0)	10 (28.6)	14 (23.3)
Missing	2 (8.0)	0 (0.0)	2 (3.3)
Eligible diagnosis, *n (%)*			
PTSD	3 (12.0)	6 (17.1)	9 (15.0)
Complex PTSD	22 (88.0)	28 (80.0)	50 (83.3)
Incorrectly Returned	0 (0.0)	1 (2.9)	1 (1.7)
Number of previous traumas, *n (%)*			
1 previous trauma	1 (4.0)	2 (5.7)	3 (5.0)
2–3 previous traumas	8 (32.0)	8 (22.9)	16 (26.7)
4–6 previous traumas	5 (20.0)	12 (34.3)	17 (28.3)
≥ 7	10 (40.0)	12 (34.3)	22 (36.7)
Missing	1 (4.0)	1 (2.9)	2 (3.3)
Number of previous treatment attempts, *n (%)*			
0 attempts	5 (20.0)	4 (11.4)	9 (15.0)
1–3 attempts	9 (36.0)	24 (69.0)	43 (55.0)
4–6 attempts	7 (28.0)	5 (14.3)	12 (20.0)
≥ 7 attempts	4 (16.0)	1 (2.9)	5 (8.3)
Missing	0 (0.0)	1 (2.9)	1 (1.7)
PTSD onset and diagnosis			
Time since PTSD onset (years), *mean (SD)*	17.92 (7.53)	13.10 (9.87)	15.29 (9.13)
Time since confirmed PTSD diagnosis (years), *mean (SD)*	11.38 (7.57)	7.37 (5.68)	8.88 (6.67)
Baseline questionnaire, *mean (SD)*			
PCL-5 score	54.88 (10.84)	58.47 (10.62)	56.95 (10.77)
WSAS score	25.28 (8.82)	23.92(7.88)	24.48 (8.24)
EQ VAS Score	48.92 (23.71)	50.80 (19.28)	50.02 (21.07)
PHQ 9 score	19.39 (5.70)	18.48 (5.88)	18.86 (5.78)
GAD 7 score	15.55 (4.63)	15.45 (4.45)	15.55 (4.63)
QPR score	43.72 (10.20)	44.46 (11.90)	44.15 (11.14)

^a^Private to Corporal

^b^Sergeant to Warrant Officer 1

### Feasibility outcomes

All the pre-ordained progression criteria were met (Table 3). The only criteria not fully met related to therapy retention. Fifty percent threshold for retention in TFCBT was achieved but RTM was almost 5% short of the threshold of 70% retention. These criteria differed by group because of the published attrition rates for these therapies [13, 15, 37]. During the trial we identified two further criteria relating to proportion of participants: (1) assessed as ineligible by the therapist and (2) not commencing therapy post-randomisation. Outcomes for these new criteria are comparable between the two therapy groups.

**Table 3:** Primary feasibility outcome measurements

Outcome	Progression criteria. Total number (unless stated as %)	Outcome in the trial%, (95% CI)
Number expressing interest	At least 180 individuals	350 Achieved
Number recruited	At least 60 participants	75 (21.43% of those that expressed interest recruited (17.43 to 26.06)) Achieved
Number randomised	At least 60 participants	60 (80% (69.23 to 87.67) of the recruited 75 participants) Achieved
Percentage of participants lost to follow up	≤ 40%	8.4% (3.45 to 18.80) Achieved
Percentage of participants deemed unsuitable for, and did not commence, therapy	New observation during trial-no progression criteria established at outset	11.7% (5.58 to 22.80)
Completeness of all outcome data for all randomised participants (Missing data point/All data points at 20 weeks)	≥ 60%	75% (62.29 to 84.49) Achieved
Completeness of PCL-5 outcome at 20 weeks	≥ 60%	75% (62.29 to 84.49) Achieved
Compliance with therapy	RTM compliance ≥ 70% TFCBT compliance ≥ 50%	60%(46.96 to 71.76) Partially Achieved

### Secondary outcomes

Sixteen participants completed outcome data at 20 weeks in the TFCBT arm, and 29 participants completed outcome data at 20–weeks in the RTM arm (Table 4). One participant in the RTM arm did not provide baseline data for some of the questionnaires and thus their 20-week data was not included in the outcome analysis.

**Table 4:** Analysis of mental health outcomes by treatment arm

Outcome	At 20 weeks		Mean change from baseline		
Questionnaire	TFCBT (*n* = 16)	RTM (*n* = 29)	TFCBT (*n* = 16)	RTM (*n* = 29)	Difference in means (RTM vs TF-CBT), 95% CI
**PCL-5, mean (SD)**	48.31 (11.98)	38.17 (17.70)	− 8.38(− 15.60 to − 1.15)	− 17.71 (− 25.80 to − 9.63)	− 9.34 (− 21.39 to 2.71)
**Percentage met PCL MCID,** ***% (95% CI)***	16.00 (5.82 to 37.00)	48.47 (32.30 to 65.25)	–	–	–
**WSAS, mean (SD)**	23.00 (9.61)	19.07 (11.50)	− 3.06 (− 7.21 to 1.09)	− 4.62 (− 8.05 to − 1.19)	− 1.56 (− 7.11 to 4.00)
**Percentage met WSAS MCID,** ***% (95% CI)***	24.00 (10.72 to 45.36)	25.71 (13.62 to 43.17)	–	–	–
**QPR, mean (SD)**	42.94 (9.56)	46.76 (13.14)	− 2.38 (− 8.26 to 3.51)	1.66 (− 3.62 to 6.93)	4.03 (− 4.33 to 12.39)
**PHQ-9, mean (SD)**	17.25 (4.80)	15.18 (7.51)	− 3.07 (− 6.27 to 0.13)	− 2.73 (− 5.43 to − 0.04))	0.34 (− 3.86 to 4.50)
**Percentage met PHQ-9 MCID,** ***% (95% CI)***	24.00 (10.72 to 45.36)	20.00 (9.58 to 37.11)	–	–	–
**GAD-7, mean (SD)**	13.50 (4.12)	11.82 (6.09)	− 3.43 (− 6.84 to − 0.02)	− 3.11 (− 5.54 to 0.68)	0.32 (− 3.86 to 4.50)
**Percentage met GAD-7 MCID,** ***% (95% CI)***	24.00 (10.72 to 45.35)	22.86 (11.56 to 40.17)	–	–	–
**EQ VAS, mean (SD)**	50.06 (21.94)	57.14 (25.15)	0.06 (− 15.81 to 15.93)	5.66 (− 4.35 to 15.66)	5.59 (− 12.30 to 23.48)

Participants in the RTM arm experienced a mean reduction of 18 points (95% CI − 25.80 to − 9.63)) on the PCL-5, a further 9-point reduction over the TF-CBT arm (95% CI − 21.39 to 2.71); however, the standard deviation was large. Furthermore, more RTM participants experienced a clinically significant reduction (48%) than in the TFCBT arm (16%). Despite the reduction in PCL-5 scores, in both arms, the mean PCL-5 remained above the PTSD diagnostic threshold of 33. Functional impairment, assessed by the WSAS, reduced in RTM and TFCBT by − 4.62 (95% CI − 8.05 to − 1.19) and − 3.06 (95% CI − 7.21 to 1.09) respectively in both groups with a slightly greater effect signal in the RTM group, as seen by a difference in mean changes of − 1.56 (95% CI − 7.11 to 4.00). Depression symptoms assessed by PHQ-9 reduced across RTM and TFCBT by − 2.73 (95% CI − 5.43 to − 0.04) and − 3.07 (95% CI − 6.27 to 0.13) respectively, with a difference in mean changes of 0.34 (95% CI − 3.86 to 4.50). In both arms, this met the MCID of − 1.7 [32]. This reduction in the MCID is also mirrored for anxiety assessed by the GAD-7 [32]. Health status improved in the RTM group only. Quality in the process of recovery only changed in the direction of improvement in the RTM group.

### Sensitivity analysis

A sensitivity analysis was undertaken by excluding further those who did not attend any therapy sessions but completed all follow-up (*n* = 1 for TFCBT arm; *n* = 1 for RTM) and those deemed unsuitable by the therapist to continue with treatment but completed all follow-up (*n* = 2 for TFCBT arm; *n* = 1 for RTM). The findings did not differ from the main analysis.

### Safety outcomes

There were no adverse events or serious adverse events reported in this trial. No participants met our safety criteria relating to PCL-5 changes between sessions or from baseline to session. The independent clinical psychologist received no contacts from participants or family members in the RTM arm.

### Qualitative—results

The qualitative study aimed to address research question 4, to explore veterans’ experiences of joining the trial, of the research procedures and therapy, and how to improve the research design for future studies with a veteran population. Fifteen veterans participated in the interviews (Table 5).

**Table 5:** Qualitative interview groups

Interview groups	*N*
RTM–completed treatment (RTM-ct)	6
RTM–stopped treatment (RTM-st)	2
TFCBT–Completed treatment (TFCBT-ct)	5
TFCBT–stopped treatment (TFCBT-st)	2
Total	15

The thematic analysis resulted in four themes as described below.

#### Experiences of joining a research programme

Most joined the study via the social media recruitment pathways. Motivations were to improve their health, to do something for the veteran community and to improve care. All participants talked about gaining trust as they were recruited.

“I was a bit apprehensive to start with. But as I got into it, I became more relaxed […] but I was made to feel relaxed and once I got into the programme, I became more confident and was able to talk more open” (Interviewee 3, TFCBT-ct).

Participants did not have a treatment preference as long as support was provided to improve their PTSD symptomatology. Their main concern was to receive treatment. Veterans expressed no concerns that the therapy was provided by a charity with some stating it as a preference.

#### Experiences of being a research participant

All participants reported positive experiences including communication, engagement, sharing information and assessments. Remaining a participant in the study, despite some not experiencing personal benefit was important and aligned with their military culture. Assessment procedures were positively viewed with the length and content being most challenging.

“I have no issues filling out a questionnaire, but ...there's a lot of words, you know, so somebody which has difficulty with reading and retention...you have to really have the focus” (Interviewee 7, RTM-st).

Whilst completing assessments were challenging, many reported that they knew they had to press on if they wanted to move forward. Participants reported formulating a relationship with their therapists and understood the importance of a therapeutic alliance.

#### Experiences of being in a research therapy

Almost all participants noticed that their symptoms reduced and noted an increased ability to handle stress.

“My wife has noticed that I'm not as snappy as I used to be, and I'm definitely thinking a lot more about how I'm reacting to certain things” (Interviewee 10, RTM-ct).

Participants discussed two key challenges; long treatment duration in TFCBT and in RTM, visualisation. PTSD symptoms remained for a few participants.

“...I would wake up… and I felt three times as bad. It was really horrible" (Interviewee 16, TF-CBT-st).

“I struggled with visualisation. I did struggle with it and I've struggled with that for a long time, but you know, we were getting there” (Interviewee 7, RTM-st).

Visualisation challenges were the main reason for withdrawing from RTM therapy.

#### Future recommendations

Regarding recruitment, interviewees advocated multiple channels including veteran specific organisations and charities, veteran communities and social media such as Facebook and Twitter. The need to identify pathways that will allow identification of ‘hard to reach’ veterans to participate was highlighted. Recommendations by their ex-commanders is important although veteran organisation-only strategies might deter some, associated with secrecy and lack of trust in the system.

*“*I think this is a big challenge because what you're talking about is sort of how we integrate studies into the wider network. So, think about how people get Uh, sort of visibility in the veterans’ community. …….For me the biggest thing will be reaching out through the different networks” (Interviewee 12, RTM-ct).

Participants stressed that retention was influenced by the outcome of the first session and therapeutic alliance. Online therapy was the only mode available consequent to the pandemic and most found this acceptable and in many cases desirable though choice was important. Several participants highlighted that a prior understanding of military ranks, hierarchical relationships within the military forces and the military system will positively affect not only processes for recruitment, participation and retention, but also the outcome and engagement with therapy.

“He (therapist) was Irish and he knew places where I was talking about. That helped, I think, and he understood what life was like at that time out there, and I think that helps.” (Interviewee 11, RTM-ct).

At the end of the research, veterans wanted their own outcomes to be communicated with their GP and the NHS so it could be used in their future care planning. It was also important for participants to know what would happen next to the research project to validate their important contribution.

## Discussion and conclusions

### Feasibility and acceptability of the trial protocol

The findings, and the quality of the data generated, show that the trial protocol was suitable to evaluate the efficacy of RTM as a potential treatment for PTSD. Within the specified pre-trial progression criteria, the trial established that (i) it is feasible to recruit veterans into a therapy trial, (ii) they will consent to randomisation into two different therapies, and (iii) they will remain in therapy and engaged with the research. Furthermore, follow-up interviews with veterans found both research therapies, and study procedures, were acceptable to participants who described overall positive experiences of taking part in the trial. Two additional progression criteria were developed during the trial, ‘*the proportion of participants deemed unsuitable for therapy by therapist’* and ‘*the proportion of participants who did not commence therapy’*. The percentage of participants who did not present for their first therapy appointments was the same in both arms at 11%. The percentage of those deemed unsuitable for therapy by their therapist differed between groups, with 20% in the TFCBT arm compared to only 5% in the RTM. Post-trial discussions with the therapists and clinical supervisors have identified likely reasons for the differences as relating to disrupted initial training for the TFCBT therapists and a protocol emphasis on PTSD diagnosis with limited attention to overall assessment of mental health and therapy readiness prior to confirmation of eligibility on the CAP 5 and ITQ post-randomisation [17, 18]. This indicates the trial protocol requires strengthening to determine eligibility prior to randomisation before it is used in the next stage of research in a definitive RCT. This strengthening will determine the potential participants’ therapy readiness alongside therapist, or therapy-related factors.

### RTM efficacy signal

An ‘efficacy signal’ was detected for RTM in veterans with complex PTSD, showing an 18-point reduction in PTSD symptoms at 20 weeks following RTM therapy. This signal exceeds the established MCID of a 10-point symptom reduction on the PCL-5 [40]. The efficacy signal for TFCBT, with an 8-point reduction, fell slightly short of the MCID. Despite the reduction in PCL-5 scores, the mean PCL-5 score remained above the threshold for losing a diagnosis of PTSD which is a score of 33 or above [17] in both arms. This may be due to the complex PTSD diagnoses of most of the participants. Nonetheless, the study identified a significant and important degree of symptom reduction and 48% of the RTM arm did lose their PTSD diagnosis compared to 16% in the TFCBT arm. Such an outcome is likely to be important for a participant population for whom over 65% have experienced four or more traumas. Neither RTM therapy nor TFBCT resulted in any adverse or serious adverse events as defined in our safety protocol.

### Strengths and limitations

The trial was delivered in the most challenging of contexts for participants, therapists, and the research team, however, the protocol adaptions that were necessitated such as online therapy and recruitment expansion across the UK ultimately strengthened the protocol for the future efficacy stages. One implication of this is that our evidence can only support the further evaluation of online RTM and not RTM delivered face-to-face. Whilst this is a strength in a resource challenged context, it may limit confidence in the main trial findings when applying them with clients offered access to, and who prefer, face to face therapy. Our trial represents the first independent evaluation of RTM outside of the team who developed the protocol in the USA. Our clinical collaborator, a third sector organisation, is not necessarily typical of veteran or mental health charities in terms of its size, scope and attention to all areas of governance which includes robust risk assessment, escalation and management protocols and procedures. Clinical impacts, therefore, may not generalise or be safely replicated in other charities. Eligibility criteria were proportionately inclusive of veterans with complex PTSD and a range of additional mental health circumstances. However, approximately 50% of those we excluded were on the grounds of complex mental health challenges, a confounding factor which would interfere with a fair assessment of the impacts of RTM. The complexity of the presenting ill-health of potential participants may have been compounded by the COVID pandemic and this picture may be different in a future main trial. Recruitment strengthening is required to ensure a more gender and ethnically diverse study population. Some further protocol development will be required prior to RTM’s evaluation in a pragmatic cost-effectiveness evaluation.

### Implications for efficacy trial

The RTM efficacy signal and the largely feasible research protocol indicates that a fully powered trial is the next appropriate step to determine the efficacy of RTM and its safe use with UK veterans and thereafter other PTSD populations. Little is known empirically of the mechanisms of RTM and research to understand how RTM achieves any effects is essential for it to be accepted as a psychological therapy. While we successfully trained charity counsellors in the use of RTM (and online RTM delivery) to optimise upscaling of RTM if efficacy is demonstrated, establishing the feasibility of training NHS healthcare workers in RTM delivery is essential.

## Conclusions

RTM therapy remains a promising psychological intervention for the treatment of PTSD and complex PTSD in military veterans. It has potential to be cost-effective compared to existing therapies. Our research protocol, which is now strengthened in areas of participant therapy readiness, can be used to underpin further evaluation.

## Data Availability

Data and materials are available on request to the corresponding author.

## Abbreviations

CAPS5: Clinician Administered PTSD scale
DSM-5: Diagnostic and Statistical Manual for Mental Disorders
EMDR: Eye Movement Desensitising and Reprocessing
EQ-5D-5L: EuroQol–5 Dimension 5 level
EQ VAS: EuroQol Visual Analogue Scale
GAD7: The General Anxiety Disorder Scale
GDPR: General Data Protection Regulation
IQR: Interquartile range
KCL: King’s College London
MCID: Minimal clinically important difference
PCL-5: Post-traumatic stress disorder checklist
PETT: Post-traumatic stress disorder experimental treatment trial
PHQ9: The Patient Health Questionnaire
PTSD: Post-traumatic stress disorder
QPR: The Quality of Process of Recovery scale
RCT: Randomised controlled trial
RTM: Reconsolidation of Traumatic Memories
SD: Standard deviation
TFCBT: Trauma-focussed cognitive behaviour therapy
WSAS: Work and Social Adjustment Scale
UK: United Kingdom of Great Britain and Northern Ireland
